# Use of a functional mobility measure to predict discharge destinations for patients admitted to an older adult rehabilitation ward: A feasibility study

**DOI:** 10.1111/ajag.12491

**Published:** 2017-12-27

**Authors:** Trish Tillson, Maheswaran Rohan, Peter J Larmer

**Affiliations:** ^1^ Auckland City Hospital Auckland New Zealand; ^2^ Department of Biostatistics and Epidemiology Auckland University of Technology Auckland New Zealand; ^3^ School of Clinical Sciences Auckland University of Technology Auckland New Zealand

**Keywords:** aged, aging, patient discharge, regression analysis, rehabilitation

## Abstract

**Objective:**

To investigate whether the discharge destination for older adults can be predicted using functional mobility as measured by the Modified Elderly Mobility Scale (MEMS), associated with demographic and primary reason for admission variables.

**Methods:**

A retrospective cohort population audit of 257 patients admitted and discharged from four tertiary older adult rehabilitation wards in a three‐month period. A number of predictor variables were considered alongside the discharge destination.

**Results:**

Multinomial statistical modelling established that MEMS prior to (P < 0.001), MEMS on completion (P = 0.009) of rehabilitation physiotherapy and primary reason for admission (P = 0.002) were significant variables to predict discharge destination. The model correctly predicted 71% of observed patient discharge destinations.

**Conclusion:**

The MEMS in conjunction with primary reason for admission was able to predict discharge destination with 71% accuracy in a heterogeneous population of older adults following rehabilitation.

**Policy Impact**

This feasibility study provides promising results to suggest that using a simple single outcome measure (the Modified Elderly Mobility Scale) is able to provide patients, their families and clinicians early and realistic information to plan discharge destination.

Practice ImpactThe findings from this feasibility study have potential to allow the appropriate level of rehabilitation to be targeted towards those older adults who will benefit the most. This also has potential cost‐saving benefit.

## Introduction

Hospitalisation rates increase as we age, and in particular, it has been found that hospitalisation rates increase shortly before older people are admitted to long‐term care 1. Functional changes often occur before hospital admission due to acute illness, and a subsequent functional decline in hospital has been identified 2, 3. Kosse et al's 4 systematic review found that early physical rehabilitation for hospitalised older adults resulted in functional benefits and reduced likelihood of discharge to residential care. In addition, studies have found that dedicated older adult wards lead to less functional deterioration on discharge 5.

The ability to predict discharge destination following rehabilitation enables the initiation of discharge planning. Early discharge planning for the older adult has been shown to reduce hospital readmissions, duration of hospital readmissions and all‐cause mortality, and to improve quality of life 6, 7, 8. Recent systematic reviews identified many social, physical and cognitive factors that predict discharge destination for stroke 9 and non‐stroke patients 10. Lindenberg et al. 11 found that diagnosis was not associated with discharge destination in a heterogeneous group of older patients undergoing rehabilitation who had previously lived at home. However, studies have found that an array of functional, cognitive and social measures used together had a high chance of predicting discharge destination 11, 12, 13. From a clinician's perspective, identifying a single, simple measure to assist in the prediction of discharge to a range of destinations would be more useful clinically than currently available tools that involve multiple assessments.

Following stroke, the acute Functional Independence Measure (FIM) independently predicts discharge to the community 14, and with mild‐moderate disability following stroke, age with admission FIM score predicts future level of care requirements 15. However, this measure is time‐consuming to complete and requires credentialing to use 16. The Elderly Mobility Scale has been established as a simple screening tool with cut‐off scores that can guide the level of care decisions within residential facilities such as rest homes and private hospitals 17. In addition, The Swedish version of the Elderly Mobility Scale has been shown to have a moderate correlation with discharge destination following hip fracture, and is predictive when utilised in combination with social, demographic and cognitive variables 18. The Modified Elderly Mobility Scale (MEMS) was developed by two alterations to the original Elderly Mobility Scale. Firstly, the timed walk was increased from 6 to 10 m with the scoring adjusted to correspond with a faster walking speed and secondly, stair‐climbing ability was added, thus increasing the utility for assessing function in older people 16. The MEMS assesses eight items of functional mobility with a possible range of 0–23, with a higher score indicating higher level of functional mobility. The items assessed are lying to sitting, sitting to lying, sit to stand, stand, gait, timed 10‐m walk, functional reach and stairs 16. The MEMS has been shown to be a valid measure of functional mobility when compared with the FIM, and highly reliable regardless of pathology or level of experience of the person administering the measure 16.

From the acute wards at Auckland City Hospital, all medically stable patients aged over 65 years who may not be safe to return to their previous residence are admitted to the older adult rehabilitation wards for multidisciplinary assessment and rehabilitation trial. The rehabilitation stay is used to determine whether the patient will be safe to discharge to their previous residence or whether an increased level of care is required. In the older adult rehabilitation wards, the MEMS is used to measure the functional mobility of all patients at admission and completion of physiotherapy, as it is quick and simple, consisting of functional tests performed during the routine assessment. Physiotherapy is completed on average three days prior to discharge from the rehabilitation ward. Discussion between the clinical team, patients and families can be difficult when there are varied opinions on appropriate discharge destination for patients. Having a simple objective measure to underpin clinical reasoning and subjective opinion could be useful particularly for junior staff, and the development of such a tool is explored in this feasibility study.

The aim of this feasibility study was to examine whether the MEMS score at admission and completion of physiotherapy, with the reason for admission and demographic variables, can predict discharge destination and therefore whether a larger study is of value.

## Methods

### Design

This study was a retrospective cohort population audit over a three‐month period.

### Data collection protocol

All patients admitted to and discharged from four tertiary older adult multidisciplinary rehabilitation wards in Auckland City Hospital, New Zealand, 4 May–31 July 2015 inclusive, were eligible for inclusion in this study. Exclusion criteria were as follows: (i) not receiving physiotherapy management; (ii) died during the stay; and (iii) insufficient data. To determine the change in patients' functional status, the MEMS was administered at the initial and final physiotherapy intervention. Demographic details, initial and final MEMS scores, primary reason for admission, length of stay and discharge destination were extracted at the end of study period (V.dT. and S.J.). Discharge destination was classified as follows: (i) home with no care (defined as not a health‐care facility and no employed home help); (ii) home with care (defined as not a health‐care facility, but with employed home help); (iii) rest home (health‐care facility providing intermittent assistance and nursing oversight); and (iv) private hospital (health‐care facility providing intensive continuing nursing care and not a rehabilitation facility) 19. Primary reason for admission was classified as follows: (i) amputation; (ii) fall (defined as fall with no fracture); (iii) medical; (iv) orthogeriatric (including fall with fracture and elective surgery); and (v) stroke.

### Ethics

Ethical approval was obtained from The Auckland District Health Board Research Review Committee (A + 6747) and the Auckland University of Technology Ethics Committee (15/199).

### Statistical analysis

Data were analysed with the statistical software R 3.2.0. The demographic and clinical characteristics were reported as number (percentage). The mean and standard deviation (SD) of MEMS at admission and completion of physiotherapy by the discharge destination were reported.

To address the categorical primary outcomes of discharge destination, multinomial statistical modelling was used to predict the discharge destination based on the variables MEMS at admission and completion of physiotherapy, age group, gender, ethnicity, length of stay and primary reason for admission. A decision was made as to which variables to include in the model based on the Akaike Information Criteria using the backward stepwise search method, and the chi‐squared likelihood method to derive a test to compare nested models 20. Graphical presentations of the probability of discharge categories based on selected variables have been provided. To assess the model validity, prediction rate by computing the number predicted by the model tallied with observed and then divided by total number of patients, has been provided.

## Results

All registered patients (*n* = 384) were considered for this study. Altogether, 127 patients were excluded: not receiving physiotherapy management (*n* = 25), deceased (*n* = 10) and insufficient data (*n* = 92), resulting in data from 257 patients. Insufficient data were primarily due to an inability to carry out the MEMS, owing to norovirus infection control procedures limiting access to the stairs, for two of the four wards for 55 days of the study period.

Patients' demographic and clinical characteristics are summarised in Table 1. Patients were predominantly female (*n* = 178, 69%), New Zealand European (*n* = 184, 72%) and over 84 years of age (*n* = 148, 57%). Approximately half of the patients were admitted with orthogeriatric conditions (*n* = 123, 48%) followed by medical conditions (*n* = 80, 31%). The majority of patients were discharged home with care (*n* = 141, 55%).

**Table 1:** Participants' characteristics

Variables	Groups	*n* (%)
Gender	Female	178 (69)
	Male	79 (31)
Age	>84	148 (57)
	75–84	77 (30)
	65–74	32 (13)
Ethnicity	New Zealand European	184 (72)
	Other European	35 (14)
	Asian	22 (8)
	Pacific	8 (3)
	Maori	5 (2)
	Other	3 (1)
Primary reason for admission	Orthogeriatric	123 (48)
	Medical	80 (31)
	Fall	42 (16)
	Stroke	10 (4)
	Amputation	2 (1)
Discharge destination	Home with no care	34 (13)
	Home with care	141 (55)
	Rest home	35 (14)
	Private hospital	41 (16)
	Transfer to another hospital	5 (2)
	Dementia care	1 (0)

The mean (SD) length of stay in the rehabilitation wards was 16.2 (9.83) days. The majority of patients improved their MEMS from admission to completion of physiotherapy (*n* = 223, 87%).

Only destination categories with more than 3% patients were considered; therefore, dementia unit (*n* = 1, <1%) and transfer to another hospital categories (*n* = 5, 2%) were removed for the rest of the analysis. Table 2 shows means of MEMS at admission and completion of physiotherapy by discharge destination.

**Table 2:** Mean (standard deviation) of Modified Elderly Mobility Scale (MEMS) at admission and completion of physiotherapy by discharge status

	Discharge destination			
	Home with no care	Home with care	Rest home	Private hospital
Mean (SD) at admission	13.9 (5.2)	10.0 (5.3)	9.0 (5.4)	3.8 (4.4)
Mean (SD) at completion of physiotherapy	19.5 (2.7)	16.5 (4.0)	14.8 (4.5)	6.3 (5.3)

Multinomial statistical modelling was used to predict discharge destination based on MEMS at admission and completion of physiotherapy, age group, gender, ethnicity, length of stay and primary reason for admission. Statistical modelling found that MEMS at completion of physiotherapy (*P* < 0.001), MEMS at admission (*P* = 0.009) and primary reason for admission (*P* = 0.002) were significant variables to predict discharge destination, and other variables such as ethnicity (*P* = 0.8), age group (*P* = 0.6), length of stay (*P* = 0.7) and gender (*P* = 0.2) were statistically not important variables to predict discharge destination.

**Figure 1:**
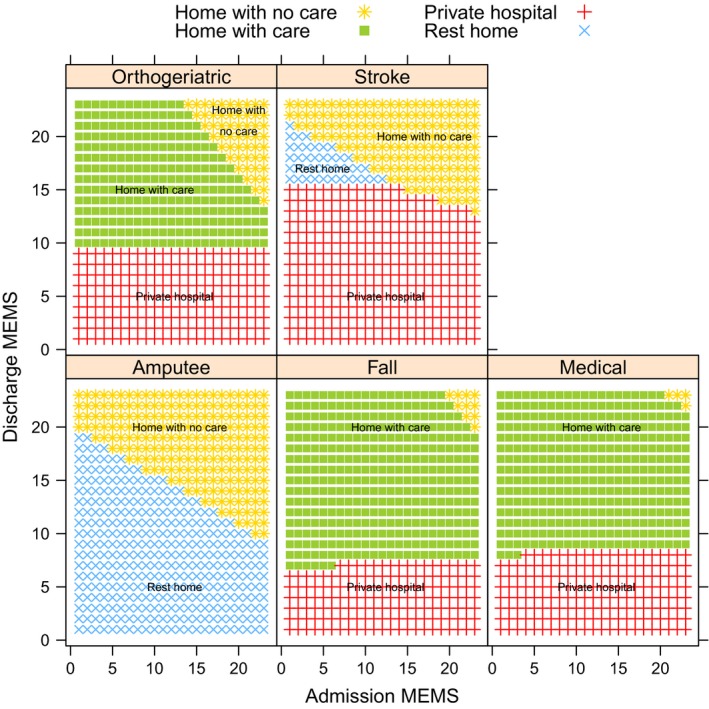
Predicted discharge destination by primary reason for admission and Modified Elderly Mobility Scale (MEMS).

Figure 1 displays the predicted discharge destination by the model. Fall patients with a discharge score 6 or below are most likely discharged to a private hospital, and those with a score above 6, to go home with care. Medical patients with a discharge score 7 or below are most likely to be discharged to a private hospital, and those above seven, home with care. For both fall and medical patients, if admission and discharge scores are more than 20, it is most likely that they will go home with no care. Orthogeriatric patients with a discharge score below 10 are most likely to be discharged to a private hospital, and those with discharge scores between 10 and 13, home with care. Those with discharge scores above 13 are likely to be discharged home with or without care depending on the admission score. Stroke (*n* = 10) and amputee (*n* = 2) patients have been included; however due to low numbers, the results should be considered with caution. Stroke patients with a discharge score <13 are likely to be discharged to a private hospital, and if discharge and admission scores are more than 15, home with no care. Amputee patients with a low score are most likely discharged to a rest home, and those with a high score, home with no care. Total patient destinations were correctly predicted in 71% of cases by the model.

## Discussion

In an effort to identify a more convenient method for clinicians to facilitate discharge planning at the completion of physiotherapy, this feasibility study investigated whether discharge destination can be predicted using the MEMS. It has not been previously established that a single functional mobility measure, the MEMS score at admission and discharge, predicts discharge destination with 71% accuracy. In contrast to other studies, the primary reason for admission was a significant predictor of discharge destination 10, 11, but age 10, 12, 13, 18, gender 13 and ethnicity 10 were not. We suggest that this study is also more generalisable than others that examined factors affecting discharge destination in discrete older adult populations, such as medical 13, stroke 9, 12, 14, 15 and hip fracture 18.

The 28% error rate was primarily because of underprediction of rest home as a destination. People previously living in a rest home would likely be discharged to there even if functionally capable of living at home following rehabilitation. Alternatively, the underprediction of rest home may imply that living in a rest home and home with care require similar functional mobility status, and destination choice is more a consequence of social and cognitive factors. This possibility is supported by the mean results of this study showing similar admission and completion of physiotherapy MEMS for both rest home and home with care (Table 2). For those patients who have an admission score of approximately 9 and likely discharge score of approximately 16, the importance of social and cognitive factors over functional ability can be made more explicit to the patient and family during decision‐making.

Clinicians such as physiotherapists, who utilise clinical reasoning to estimate likely functional mobility improvement when goal setting, are well placed to use the results from this predictive tool to underpin their clinical reasoning during family and multidisciplinary discharge planning meetings. The ability to predict likely discharge destination for older adults admitted to rehabilitation wards has a number of practical benefits. If a move to a new environment is likely to be necessary, an early family meeting allows patients and families time to adjust to the change. It also helps avoid prolonged admissions due to time taken to find suitable residential care and for a place in the facility to become available. If the older adult is likely to go home, it allows time for practical preparations such as arranging cleaning or implementing recommendations to reduce risk, such as installing rails on stairs or reducing clutter. For the rehabilitation team, knowing likely discharge destination facilitates referral for assessment for carers or residential placement, and for the patient going home, enables timely organising of required compensatory equipment. In addition to the practical benefits of the early planning of discharge, the literature demonstrates considerable quality of life and health economic benefits, with a sustainable discharge leading to a reduction in hospital readmission, length of hospital readmission stay and all‐cause mortality 6, 8.

Data were collected from rehabilitation wards in one hospital and therefore may not be generalisable to other older adult rehabilitation settings. Although the risk of selection bias was reduced with a cohort design, as previously discussed there were insufficient data from 24% of patients who underwent rehabilitation during the study period. To enhance clinician ability to initiate earlier discharge planning, it would be useful in future to establish whether a MEMS assessed at one week, being the half way point of the average two‐week older adult rehabilitation stay, is predictive of discharge destination.

## Conclusion

With an ageing population and associated increased demands on health‐care resources, this is a crucial time in health care when timely, safe and sustained discharge is a priority. The findings of this feasibility study demonstrate that discharge destination can be predicted by a simple, clinically convenient method, the MEMS, when considered in conjunction with primary reason for admission. Predicting destination underpins discharge planning, resulting in increased well‐being of the older adult and subsequent health economic benefits for society.
